# Albumen in Urine—Its Value in Diagnosis

**Published:** 1854-01

**Authors:** 


*Albumen in Urine—Its Value in Diagnosis.* By CHAS. A. LEE, M. D., &c. In a Letter to the Editor.

MY DEAR DR.—It has become very common, of late, with certain practitioners, if they detect the presence of albumen in the urine, by heat or nitric acid, to pronounce, at once, an unfavorable diagnosis, on the ground that the patient is laboring under albuminuria, or Bright's disease of the kidney. Many persons have been unnecessarily alarmed, and their disease, whatever it may have been, greatly exasperated by the influence which such an opinion has had upon the mind, and, through that, upon the disease. While I have no doubt that the danger of that disease (albuminuria) has been greatly exaggerated, I have still less, that a false diagnosis is often made, and that patients are told that they labor under this disease, when there is no good foundation for such belief. There are many circumstances under which albumen exists in the urine, independent of every structural change in the urinary organs, and these circumstances should be more generally known. Simon tells us that albumen is often found in the urine of persons in *perfect health*, and though this statement, perhaps, needs confirmation, by more extensive experiments than have, as yet, been made on healthy urine, there is no good reason to doubt its correctness. Observation has been hitherto chiefly limited to diseased urine, or urine secreted during some other disease, and I think there is no good reason to doubt that albumen will yet be found to be not an unfrequent ingredient in the urine of perfect health. At any rate, we know that the urine of pregnant females often contains albumen, especially in first pregnancies, and in cases of twins, owing probably to unusual congestion, produced by the pressure of the impregnated uterus, and temporary congestion from any cause, even short of disease, will be likely to produce the same result.

Since I have made it a rule to examine the urine in all diseases, I have often met with albumen in it in acute diseases generally, as well as some chronic ones, independent of any disease of the kidneys themselves, and I believe it will yet be found, that in a majority of acute diseases, albumen, in greater or less quantity, will be found during some period of them; to febrile and inflammatory affections, these periods will be about the commencement of the febrile excitement, and just after the crisis, when convalescense is about to be established. This is well observed in all the exanthematous fevers, especially measles, scarlatina and small-pox, and the presence of albumen in these cases may actually be regarded as a favorable symptom. It neither indicates the presence of organic disease in the urinary organs nor any tendency thereto. It is the result of a series of changes, which seem essential to a restoration to health, and is of no more consequence than the desquamation and exfoliation of the cuticle. I do not mean to say that albumen is always present in these diseases in their early stages, but, as a general rule, it will be found during the desquamative stage. Martin Solon says, "in twenty-two out of twenty-three cases," according to his observation; and Simon says it is "commonly" found. It may not last for more than two or three days; for the most part, it does not, and will generally be found to be associated with a considerable amount of kidney epithelium, but no fibrinous casts. The quantity of urine secreted will not vary far from the normal standard, and in other respects it will be nearly natural. It is possible that, in some epidemics of scarlatina, as stated by Chrisitison, albumen may not be found, as a general rule, in the urine, just as we find some one particular feature or symptom absent, but these varying results may, in some cases, be owing to want of care in making the observation and experiments. The examination of the urine should be made daily, or several times a day, and then the treatment may have considerable influence in preventing its presence in the urine, as the warm bath, and other revulsive means, which determine the blood to the surface, and thus relieve renal and other internal congestions, and then it should be recollected that the presence of albumen in the urine is often limited to a short time, as one day, or a few hours, and unless we daily examine it, we shall be liable to be deceived. These remarks will apply also to small-pox and measles. I have observed, also, and the same observation has often been made by others, that during the acute stage of *erysipelas* the urine will be found highly albuminous, as well as during the stage of desquamation, when it may last for several days, and contain an abundance of epithelium scales. This state of the urine is more common, it is true, in idiopathic erysipelas, where the disease involves a considerable extent of surface, but it will probably be met with more or less frequently in every form; but then the urine must be frequently examined. So, too, during the stage of reaction in *cholera*, the urine will generally be more or less coagulable; at the same time the urine will be absent, or in small quantity, while there will be present biliary coloring matter and epithelium scales. The presence of renal epithelium shows the occurrence of desquamation, and probably, as Dr. Bigbie has suggested, the passage of the albumen from the blood is closely associated with, or depends on, this process; and that too, independent of renal congestion. But this yet remains to be proved. It must be admitted, however, that we often meet with albumen in the urine in scarlatina, where there is no other evidence of renal congestion, as lumbar pain or uneasiness, or diminished urinary secretion. In the dropsy that follows scarlet fever, we find albumen in the urine in a large majority of cases, while, at the same time, the urine is scanty in quantity, sometimes tinged with blood, and usually charged with exudative corpuscles, epithelium, and fibrinous casts of the small tubes. Here there can be no reasonable doubt of the presence of renal congestion, for we have, in connection with the diminished secretion, pain and a sense of weight in the lumbar region, with occasional vomiting, and whether organic renal disease may not grow out of such congestion, when severe or long continued, is a question to be determined by future observations.

In variola, in the few cases in which the urine has been tested, albumen has occasionally been discovered in the early stage; also, in the suppurative or critical stage, but more frequently in the stage of desquamation, and not unfrequently tinged with blood, with amorphous or crystaline urates and epithelium.

It is now a well known fact, that albumen often exists in the urine in *pneumonia*, about the period of the resolution of the disease. Simon observed it in twenty-two out of twenty-four cases, at this period, and sometimes during the inflammatory stage. I believe it is, however, generally connected with the absorption of the pulmonary exudation, and it may, perhaps, be, as Schonlein has suggested, that the kidneys act as the chief emunctory for the escape of such matters from the blood, which are chiefly albuminous, although the urine also abounds with lithates in such cases. Albumen has also been detected in the urine in *pericarditis*, *endocarditis*, *pleurisy*, *carditis*, *peritonitis*, &c., and after the application of blisters.

In regard to chronic diseases, there is good reason to believe that, in most organic and structural diseases of the kidneys, the urine will be albuminous, though such is not always the case, even in Bright's disease, at certain periods. It may, however, be stated, as a general rule, that in a majority of organic diseases, whether of the abdominal or pelvic viscea, the urine will, at times, coagulate by heat or acid. I have known this happen so often in a hepatitis (recent) carcinoma of the liver, and enlargements of the spleen, that I have come to regard it as a common occurrence. In some cases where albuminous urine isassociated with dropsy, as ascites it is possible that the urine may become albuminous from the influence of stimulating and irritant diuretics, as squill, turpentine, juniper, cantharides, &c. In all cardiac or other affections, calculated to produce congestion of the portal system, and a reflux of blood through the mesenteric and renal veins, we may expect to find more or less albumen, at times, in the urinary secretion; and the same remark will apply to pulmonary diseases, as phthisis, pneumonia. The influence of stimulating diuretics in causing coagulable urine has long been known, but it is too often lost sight of in treating those affections in which albuminous urine is apt to occur, independent of their action, as Bright's disease; any person can easily test this point by taking a few doses of cantharides, and then test the urine; or juniper, which also tends to produce temporary renal congestion, will produce the same effect. So, also, will blisters, when they cause stranguary, and sometimes when they do not. In dyspeptic cases, too, the urine will often be found albuminous, and in temporary fits of indigestion produced by eating pastry, hot rolls, &c., the same happens. Recent observations in the Royal Infirmary of Edingburgh prove that albumen is found in the urine in a majority of cases of typhus fever, about the sixteenth day of the disease, and continuing four or five days; also, in a vast majority of cutaneous affections, where a large portion of the cutaneous surface is affected.

These remarks will suffice to show that we are, as yet, ignorant, in a great measure, in regard to the diagnostic value of this sign. It is significant, doubtless, of something, but what that something may be, we cannot always determine. We generally connect it, and no doubt truly, with renal congestion, temporary or permanent; but, whether that congestion be associated with structural changes, we have no means of deciding. That it often is, we know, and we know with the same certainty that it often is not—whence then the propriety, in chronic cases, in basing an unfavorable prognosis on this alone? We do not proceed thus in other cases, and I see no good reason why we should do so in this. The fact is, that renal pathology is yet in its infancy; it is only within a few years that any considerable attention has been paid to the constitution of the urine, or that chemical and microscopicscience have contributed their aid to elucidate renal diseases. I no doubt have that many anomalous cases of disease, whose pathology has hitherto been obscure, and unknown, will yet be found to have their origin in the retention of *urea* in the blood, and this is only to be ascertained by a careful and repeated analysis of the urine, with due regard to all the circumstances which modify the condition of this fluid. I have recently treated some cases of obstinate head-ache, on this hypothesis, with diuretics, and with complete success; colchicum and turpentine, with hydriodate of potash, increase the quantity of lithates and urea in the urine, and thus relieve the system of a *materies morbi*, which is productive of a great number of anomalous and painful symptoms, including, probably, rheumatism and gout.

But I did not sit down to write you an essay or monograph, but merely to throw out some hints in regard to albuminous urine, and to show that it is not so grave a sign as some pathologists are disposed to regard it, and much remains to be learned, and many more observations to be made before we shall be able to understand its true diagnostic value.

Truly yours, CHAS. A. LEE.

